# Cancer symptom cluster in hospitalized women with breast cancer: a descriptive observational study

**DOI:** 10.1590/0034-7167-2024-0091

**Published:** 2025-04-28

**Authors:** Roberto Júnio Gomes Silva, Wesley Rocha Grippa, Raphael Manhães Pessanha, Júlia Anhoque Cavalcanti Marcarini, Luiz Cláudio Barreto Silva, Naira Santos D’Agostini, Luís Carlos Lopes-Júnior

**Affiliations:** I Universidade Federal do Espírito Santo. Vitoria, Espírito Santo, Brazil

**Keywords:** Signs and Symptoms, Concurrent Symptoms, Breast Neoplasms, Medical Oncology, Oncology Nursing, Signos y Síntomas, Síntomas Concomitantes, Neoplasias de la Mama, Oncología Médica, Enfermería Oncológica

## Abstract

**Objectives:**

to describe and analyze the most prevalent cancer symptom clusters as well as their intensity, discomfort and clustering in hospitalized women with non-metastatic malignant breast cancer.

**Methods:**

a descriptive observational study, with 100 women hospitalized with non-metastatic breast cancer, recruited at a reference oncology center in southeastern Brazil between June 2022 and March 2023. To analyze cancer symptom clusters, the hierarchical method was used.

**Results:**

fifty-one patients were in stage I of the disease, 13 women in stage II, and 36 women in stage III. The most frequent histological type was ductal carcinoma *in situ* (38%), followed by invasive carcinoma (33%). The most prevalent cancer symptoms were pain (67%), lack of energy (63%), worrying (62%), and difficulty sleeping (57%), constituting a neuropsychological symptom cluster.

**Conclusions:**

the neuropsychological symptom cluster (pain-lack of energy-worrying-difficulty sleeping) was the most prevalent in women hospitalized with non-metastatic malignant breast neoplasm.

## RESUMEN

**Objetivos:**

describir y analizar los síntomas oncológicos más prevalentes, así como su intensidad, malestar y agrupación en mujeres hospitalizadas con cáncer de mama no metastásico.

**Métodos:**

estudio observacional descriptivo, con 100 mujeres hospitalizadas con cáncer de mama no metastásico, reclutadas en un centro de referencia en oncología del sudeste de Brasil entre junio de 2022 y marzo de 2023. Para analizar clusters de síntomas oncológicos se utilizó el método jerárquico.

**Resultados:**

51 pacientes se encontraban en el estadio I de la enfermedad, 13 mujeres en el estadio II, 36 mujeres en el estadio III. El tipo histológico más común fue el carcinoma ductal in situ (38%), seguido del carcinoma invasivo (33%). Los síntomas oncológicos más prevalentes fueron dolor (67%), fatiga (63%), preocupaciones (62%), trastornos del sueño (57%), constituyendo un cluster de síntomas neuropsicológicos.

**Conclusiones:**

el cluster de síntomas neuropsicológicos (dolor-fatiga-preocupaciones-alteración del sueño) fue el más prevalente en mujeres hospitalizadas con cáncer de mama no metastásico.

## INTRODUCTION

Malignant breast neoplasm is considered the most common tumor and the one with the highest mortality rate in women, not only in Brazil, but also worldwide^(1,2)^. According to data from the Brazilian National Cancer Institute (In Portuguese, *Instituto Nacional do Câncer* - INCA), this type of neoplasia is responsible for 28% of new annual cases of all types of cancer^(3)^. It is estimated that, in Brazil, one in every 12 women will develop this pathology over the course of their life^(3)^.

Patients with malignant breast neoplasia experience a series of symptoms caused by the tumor microenvironment itself and also associated with the side effects of the antineoplastic treatment used^(4-6)^. This interrelated symptom cluster is called a cancer symptom cluster, which can be defined as two or more symptoms that are related to each other and that occur together, without necessarily being related to other clusters, with the relationship between symptoms in the same cluster being stronger than the relationship with different clusters^(7-10)^.

Symptoms experienced by breast cancer patients can be physical, mental and psychological in origin, and include cancer-related lack of energy, pain, difficulty sleeping, anxiety, depression and decreased cognitive function^(7,8,11)^, which are generally associated with antineoplastic treatments or the tumor itself^(7,8)^. Other very common symptoms are nausea, vomiting, loss of appetite, diarrhea and/or constipation, dry mouth and lack of appetite, which directly impact patients’ nutritional status, generating deficits in food consumption, with consequent weight loss and loss of muscle mass^(7,8,11)^.

The first studies involving cancer symptom clusters came from experiments with mice subjected to the induction of infectious conditions and the injection of pro-inflammatory cytokines, triggering inflammatory conditions in these animals, which culminated in the phenomenon of “sickness behavior”^(6,7)^. Sickness behavior was established in 1992 to designate a set of behavioral changes that occurred with diverse pathological and inflammatory processes, such as the apparent loss of interest of these animals in daily activities, such as searching for food, decreased sexual interest and social interaction^(6,7,12)^.

Studies in animals and humans have shown that cytokine infusion also induces the phenomenon of sickness behavior^(6-8,12)^. In fact, similarly to the phenomenon of sickness behavior, presented by laboratory animals, in humans, especially in cancer patients, cases of cancer pain, cancer-related lack of energy, difficulty sleeping, anxiety and depression associated with high levels of expression of pro-inflammatory cytokines have been observed^(6-8,11,12)^, evidencing the close relationship between the pattern of sickness behavior seen in experiments with animals and the pattern of cancer symptom clusters reported by cancer patients^(8,12)^.

Scientific evidence consistently supports that common biological mechanisms may underlie the interaction between the nervous, endocrine and immune systems, which orchestrate a set of responses capable of installing behavioral and physiological changes in both the animal and human organisms^(6-8,11,12)^. Furthermore, studies that encompass disease behavior in animal experiments, as well as cancer symptom clusters in humans, support the hypothesis that pro-inflammatory cytokines are one of the biological mechanisms underlying the emergence of these symptom clusters, especially the release of IL-1β, IL-6, IL-8, TNF-α, IL-12p70 and IFN-y^(6-8,11)^. This pattern of expression of pro-inflammatory cytokines results, for instance, in cancer symptom clusters in cancer patients, composed of cancer-related lack of energy, depressed mood, depression, difficulty sleeping and increased sensitivity to pain^(6-8,11,12)^.

In the tumor microenvironment, cytokines stimulate the host response to control cellular stress and reduce cellular damage^(4,6,11)^. Thus, alterations in cytokines and other neuroimmunological processes may be critical for producing cancer symptom clusters in cancer patients and may potentially be considered as targets for the prevention and treatment of these symptoms^(6-8,10-13)^. The cytokines IL-1, IL-6 and TNF-α are implicated, for instance, in the pathophysiology of cancer-related lack of energy and difficulty sleeping^(6,11)^. A study conducted with women with breast cancer, at different stages of the disease, showed that high concentrations of the IL-1β receptor antagonist (IL-1βra) were associated with high levels of cancer-related lack of energy^(14)^.

The numerous and complex cancer symptoms that can be presented by patients with breast cancer negatively impact their health-related quality of life^(4)^. Furthermore, these symptoms are associated with worse prognoses, including low survival and low adherence to cancer treatment^(4,12)^. The importance of assessing and intervening in such symptom clusters, not only in terms of patient survival, but, above all, in terms of quality of life during and after treatment, is well established and is currently an integral part of the pillars of research in oncology and, particularly, in oncology nursing^(12,13)^. The description and analysis of these symptoms and their clustering become crucial to guide the actions of multidisciplinary and interprofessional teams in approaching personalized care for these patients.

## OBJECTIVES

To describe and analyze the most prevalent cancer symptom clusters as well as their intensity, discomfort and clustering in hospitalized women with non-metastatic malignant breast cancer.

## METHODS

### Ethical aspects

The study was approved by the *Universidade Federal do Espírito Santo* Health Sciences Center Research Ethics Committee, on May 10, 2022, under Certificate of Presentation for Ethical Consideration (In Portuguese, *Certificado de Apresentação para Apreciação Ética* - CAAE) and approval Opinion, in accordance with guidelines and regulatory standards for research involving human beings in Brazil, established by Resolution 466/2012. It is noteworthy that all patients were approached and informed about the research content and objectives, expressing their agreement to voluntarily participate in the study by signing the Informed Consent Form before the beginning of the research, in two copies, one of which was given to participants and the other remained with the main researcher. It is emphasized that research participants’ anonymity and privacy were respected.

### Study design, period and location

This is a descriptive observational study with a quantitative approach, guided by STrengthening the Reporting of OBservational studies in Epidemiology (STROBE) recommendations.

The study was conducted in Inpatient Sector A of *Afecc-Hospital Santa Rita de Cássia* (HSRC), located in the capital Vitória, Espírito Santo, Brazil. HSRC is a philanthropic entity, created in 1970, the only High Complexity Oncology Care Center (In Portuguese, *Centro de Assistência de Alta Complexidade em Oncologia* - CACON) in the state and recognized throughout Espírito Santo as a reference in cancer treatment. The hospital maintains a philanthropic character, and dedicates 60% of its care to Brazilian Health System patients. For this reason, it also receives patients from other states, such as southern Bahia, eastern Minas Gerais and northern Rio de Janeiro. It has a structured Hospital Cancer Registry (HCR) that has been operating since 2000, with its databases sent annually to the *Sistema Integrado do Registro Hospitalar de Câncer* (SISRHC)^(15)^.

### Population, inclusion and exclusion criteria

Based on the latest estimate from INCA in Brazil for the three-year period 2023-2025, the incidence of female breast cancer is 74 thousand cases for Brazil, with 84.46/100 thousand cases expected for the southeast^(3)^. The service caseload in Inpatient Sector A of HSRC (data collection site) was 225 cases hospitalized with a diagnosis of malignant breast neoplasm in different phases of antineoplastic treatment in 2019 and 182 cases in 2020, configuring an average of 204 cases/year in the two-year period. In our calculation, we considered only the prevalence of cases of malignant breast neoplasms in 2019, aiming to reduce the bias of the new coronavirus pandemic, which greatly altered the flow of patients and hospitalizations in 2020.

From the formula used to calculate the sample size, which is n = N.Z2. p.(1-p) / Z2.p.(1-p) + e2.N-1, where n means the calculated sample, N, the population, Z, the normal variable, p, the real probability of the event, and the sampling error^(16)^, and considering the population of patients diagnosed with breast cancer at HSRC in 2019 (n = 225), based on the International Classification of Diseases (ICD-10) C50 (Malignant neoplasm of the breast) and setting α at 5% (sampling error), with a 95% confidence level and a β of 0.2 (providing 80% test power), with a minimum percentage of 12%, the sample n of this research was obtained equal to 95 patients.

Female patients over 18 years of age, with a pathological diagnosis of breast cancer in stages I, II or III, based on ICD-10 C50 (Malignant neoplasm of the breast), at any stage of antineoplastic treatment were included in this study.

Patients in stage IV, in exclusive palliative care, who were hospitalized for reconstructive surgery or due to clinical complications unrelated to breast cancer were excluded. It should be noted that there were no losses or patient refusals in the present study.

### Study protocol

Data collection took place between June 2022 and March 2023. Research participants who met the eligibility criteria were recruited upon admission to Inpatient Sector A of HSRC. A sociodemographic and clinical questionnaire was prepared by the researchers, which was structured into three components, namely: I) anamnesis (history of oncological disease, previous history and cardiovascular risks, family history, lifestyle habits, nutrition, physical activity practice); II) general and specific physical examination consisting of vital signs, head/neck/neurological examination, chest/lungs/heart, and abdomen; III) laboratory tests that were part of the hospital routine and were accessed from patients’ own medical records (blood count and capillary blood glucose). The sociodemographic variables included in the questionnaire were age, race, origin, income, education, occupation, marital status, number of children, tobacco and alcohol consumption. The clinical variables that comprised the questionnaire were diagnostic classification, histological type, tumor-node-metastasis, stage, previous diseases and previous surgeries. It is important to note that all data were collected via a questionnaire administered at the bedside by an oncology nurse (first author of this research). Furthermore, we emphasize that permission was obtained from hospital management and from Inpatient Sector A of HSRC regarding data collection from patients’ medical records.

To assess cancer symptom clusters, the Memorial Symptom Assessment Scale (MSAS) was used^(17)^. MSAS was developed by Portenoy *et al*. (1994)^(18)^. The MSAS is an instrument that helps detect and monitor multiple symptoms in cancer patients^(18)^, and is used worldwide in the oncology field. The MSAS combines different symptoms, with their respective degrees of frequency, intensity and discomfort. It is a self-report instrument in which patients assign a numerical value from 1 to 4 points for the frequency and intensity of 32 symptoms and from 0 to 4 for the degree of discomfort experienced during the last week. Furthermore, it is divided into subscales, which assess psychological symptoms (PSYCH), with six items, and physical symptoms (PHYS H and PHYS L), with 26 items^(18)^.

We used the validated version for Brazilian Portuguese, which showed that the scale reliability was satisfactory in test-retests^(19)^. The Kappa values obtained for each item of the scale were adequate, with the highest item being 0.96 and the lowest being 0.69. The Kappa of the subscales was also assessed, being 0.84 for high-frequency physical symptoms, 0.81 for low-frequency physical symptoms, 0.81 also for psychological symptoms, and 0.78 for the General Distress Score. The authors concluded that the high levels of reliability estimated allow us to state that the process of measuring the MSAS items was adequate^(19)^.

### Analysis of results, and statistics

The data were tabulated in a Microsoft Excel^®^ spreadsheet and analyzed using the Statistical Package for the Social Sciences (SPSS) version 28.0. The study results were presented in descriptive statistics, containing absolute frequencies and percentages for all variables studied. The variables age and those related to the MSAS were presented using mean and standard deviation. For symptom cluster analysis, the hierarchical method was used^(20)^. Hierarchical cluster analysis is an exploratory statistical technique that groups observations into clusters or groups based on their similarities or dissimilarities. The result of this clustering was presented in the form of a dendrogram, which consists of the visual representation of these clusters, in which the most similar groups are joined first and the most dissimilar are located at opposite ends of the dendrogram. The hierarchical method minimizes the total variance within the clusters, and is often used in cluster analysis^(20)^. Euclidean distance was used as a measure of similarity between symptoms in the hierarchy, seeking to group them according to their proximity in a multidimensional space based on the attributes of frequency, intensity and discomfort. This approach allowed the identification of groups of related symptoms, providing a hierarchical understanding of the relationships between MSAS symptoms. The significance level used in all analyses was 5%.

## RESULTS

The study included 100 hospitalized women diagnosed with non-metastatic breast cancer. The sample demographic and clinical characteristics are presented in Table 1 and Table 2, respectively. Participants’ mean age was 59.15 years, and standard deviation was 10.27 years. Half of the sample was represented by brown women (50%), married (50%), with two or more children (59%), and with a complete elementary education level (44%). The majority lived in the Metropolitan Region of the state of Espírito Santo (81%), were housewives (43%), earned one minimum wage (70%), were non-smokers (78%) and did not consume alcohol (86%) (Table 1).

**Table 1:** Sociodemographic and lifestyle characteristics of hospitalized women with non-metastatic breast cancer, Vitória, Espírito Santo, Brazil, 2023

Variables	*n*	%
Age (in years)		
Mean (standard deviation)	59.15 (10.27)	-
Age range		
< 50 years	15	15.00
50-64 years	53	53.00
≥ 65 years	32	32.00
Self-reported color		
White Black	38 12	38.00 12.00
Brown	50	50.00
Health region		
Metropolitan Region	81	81.00
Central-North Region	14	14.00
South Region	5	5.00
Source of income		
Retiree	56	56.00
Pensioner	9	9.00
Employee	17	17.00
No income	18	18.00
Income		
< 1 minimum wage	7	7.00
1 minimum wage	70	70.00
2 minimum wages	16	16.00
≥ 3 minimum wages	7	7.00
Education		
Illiterate	9	9.00
Elementary school	45	45.00
High school	32	32.00
Higher education	14	14.00
Occupation		
Housewife	43	43.00
Maid	22	22.00
Other^*^	35	35.00
Marital status		
Single	21	21.00
Married	50	50.00
Widower	15	15.00
Divorced	14	14.00
Children		
None	16	16.00
1	25	25.00
≥ 2	59	59.00
Do you use tobacco?		
No	93	93.00
Yes	7	7.00
Have you ever smoked?		
No	78	78.00
Yes	22	22.00
Do you drink alcohol?		
No	86	86.00
Yes	14	14.00

* *Other: hairdresser, seamstress, teacher, tour guide, cook.*

**Table 2:** Clinical profile of hospitalized women with non-metastatic breast cancer, Vitória, Espírito Santo, Brazil, 2023

Variables	*n*	%
Current cancer diagnosis		
Malignant neoplasm of the breast	37	37.00
Malignant neoplasm of the central portion of the breast	20	20.00
Malignant neoplasm of the upper-inner quadrant of the breast	19	19.00
Malignant neoplasm of the lower-inner quadrant of the breast	2	2.00
Malignant neoplasm of the upper-outer quadrant of the breast	2	2.00
Malignant neoplasm of the lower-outer quadrant of the breast	6	6.00
Malignant neoplasm of the axillary tail of the breast	7	7.00
Malignant neoplasm of the breast with an invasive lesion	7	7.00
Histological type		
Invasive carcinoma	33	33.00
*Ductal carcinoma in situ*	38	38.00
*Lobular carcinoma in situ*	29	29.00
Tumornodulemetastasis		
T1N1M0	8	8.00
T1N2M0	13	13.00
T2N1M0	14	14.00
T2N2M0	29	29.00
T3N1M0	21	21.00
T3N2M0	6	6.00
T4N2M0	5	5.00
T4N3M0	4	4.00
Stage		
I	51	51.00
II	13	13.00
III	36	36.00
Hypertension		
No	71	71.00
Yes	29	29.00
Dyslipidemia		
No	96	96.00
Yes	4	4.00
Diabetes Mellitus		
No	87	87.00
Yes	13	13.00
Previous surgery		
No	36	36.00
Yes	64	64.00

Among the 100 women with non-metastatic breast cancer, 51 patients were in stage I of the disease, 13 women in stage II, and 36 women in stage III. The most frequent histological type was ductal carcinoma *in situ* (38%), followed by invasive carcinoma (33%). Among the study participants, 29% of women in the sample were hypertensive; 13% had diabetes; and 64% had already undergone some surgery. These data are detailed in Table 2.

Table 3 presents the frequency, intensity and discomfort of oncological symptoms in women hospitalized with non-metastatic breast cancer. The most prevalent symptoms in these patients were, respectively, pain (67%), lack of energy (63%), worrying (62%) and difficulty sleeping (57%), assessed using the MSAS.

**Table 3:** Oncological symptoms of hospitalized women with non-metastatic breast cancer according to frequency, intensity and discomfort, Vitória, Espírito Santo, Brazil, 2023

MSAS symptoms		Prevalence		Frequency	Intensity	Discomfort
		*n*	%	M ± SD	M ± SD	M ± SD
1	Difficulty concentrating	27	27.00	2.59 ± 0.69	2.30 ± 0.54	3.11 ± 1.48
2	Pain	67	67.00	2.94 ± 0.87	3.09 ± 0.95	3.43 ± 0.97
3	Lack of energy	63	63.00	2.97 ± 0.76	2.78 ± 1.04	2.98 ± 1.20
4	Cough	32	32.00	3.19 ± 0.82	2.25 ± 0.88	2.66 ± 1.36
5	Feeling nervous	48	48.00	2.73 ± 0.89	2.60 ± 0.84	3.29 ± 1.07
6	Dry mouth	51	51.00	2.86 ± 0.98	2.53 ± 1.05	2.55 ± 1.23
7	Nausea	37	37.00	2.68 ± 0.67	2.59 ± 0.72	3.08 ± 1.19
8	Feeling drowsy	39	39.00	3.18 ± 0.56	2.79 ± 0.83	3.13 ± 1.38
9	Numbness/tingling in hands/feet	40	40.00	2.63 ± 1.00	2.70 ± 1.26	2.80 ± 1.20
10	Difficulty sleeping	57	57.00	3.18 ± 0.68	3.19 ± 0.77	3.23 ± 0.96
11	Feeling bloated	40	40.00	3.05 ± 0.64	2.83 ± 0.68	3.03 ± 0.99
12	Problems with urination	1	1.00	2.00	2.00	1.00
13	Vomiting	18	18.00	2.11 ± 0.58	2.33 ± 0.77	3.00 ± 0.84
14	Shortness of breath	26	26.00	2.88 ± 0.71	3.00 ± 0.69	2.77 ± 1.07
15	Diarrhea	36	36.00	2.39 ± 0.96	2.03 ± 0.51	2.86 ± 0.93
16	Feeling sad	38	38.00	3.32 ± 0.87	3.32 ± 0.90	3.50 ± 0.83
17	Sweats	42	42.00	2.38 ± 0.54	2.38 ± 0.66	2.93 ± 0.95
18	Worrying	62	62.00	3.16 ± 0.85	2.82 ± 0.98	2.85 ± 1.51
19	Problems with sexual interest or activity	18	18.00	3.06 ± 0.64	2.44 ± 0.51	2.83 ± 1.76
20	Itching	17	17.00	1.88 ± 0.60	1.82 ± 0.64	1.71 ± 1.31
21	Lack of appetite	43	43.00	2.91 ± 0.81	2.58 ± 0.73	2.33 ± 1.21
22	Dizziness	32	32.00	2.41 ± 0.50	2.22 ± 0.71	2.81 ± 1.18
23	Difficulty swallowing	26	26.00	2.85 ± 0.73	2.38 ± 0.80	2.69 ± 1.38
24	Feeling irritable	36	36.00	2.69 ± 0.52	2.64 ± 0.59	2.83 ± 0.91
25	Mouth sores	15	15.00	1.80 ± 0.77	1.80 ± 0.94	2.13 ± 1.30
26	Change in the way food tastes	37	37.00	3.14 ± 0.89	2.68 ± 0.78	2.76 ± 1.36
27	Weight loss	42	42.00	2.81 ± 0.94	2.95 ± 1.01	2.40 ± 1.71
28	Hair loss	43	43.00	3.35 ± 0.90	3.16 ± 0.90	3.51 ± 1.01
29	Constipation	43	43.00	2.67 ± 0.78	2.60 ± 0.90	2.53 ± 1.42
30	Swelling of arms/legs	19	19.00	3.05 ± 0.85	2.79 ± 1.18	2.68 ± 1.60
31	“I don’t look like myself”	31	31.00	3.10 ± 0.98	2.90 ± 1.04	3.26 ± 1.12
32	Changes in skin	12	12.00	2.50 ± 0.67	2.42 ± 0.79	1.75 ± 0.87

*MSAS - Memorial Symptom Assessment Scale (95% Confidence Interval); M - mean; SD - standard deviation.*

Chart 1 presents the hierarchy of clusters obtained through MSAS according to the hierarchical cluster method assumptions, as described in the method section.

**Chart 1:** Identification of cancer symptom clusters from the Memorial Symptom Assessment Scale of women with non-metastatic breast cancer hospitalized according to the Ward method, Vitória, Espírito Santo, Brazil, 2023

Cluster	MSAS symptoms
**1**	Diarrhea
	Sweats
	Dizziness
	Lack of appetite
	Feeling irritable
**2**	“I don’t look like myself”
	Feeling sad
	Hair loss
	Change in the way food tastes
	Feeling bloated
	Feeling nervous
	Nausea
**3**	Pain
	Difficulty sleeping
	Lack of energy
	Worrying
**4**	Weight loss
	Feeling drowsy
	Constipation
**5**	Vomiting
	Shortness of breath
	Problems with urination
	Changes in skin
	Swelling of arms/legs
	Difficulty concentrating
	Problems with sexual interest or activity
**6**	Dry mouth
	Numbness/tingling in hands/feet
	Cough
	Difficulty swallowing
	Itching
	Mouth sores

*MSAS - Memorial Symptom Assessment Scale.*

Neuropsychological symptom cluster 3 (pain, lack of energy, worrying and difficulty sleeping) was the most prevalent in the sample.

**Figure 1:**
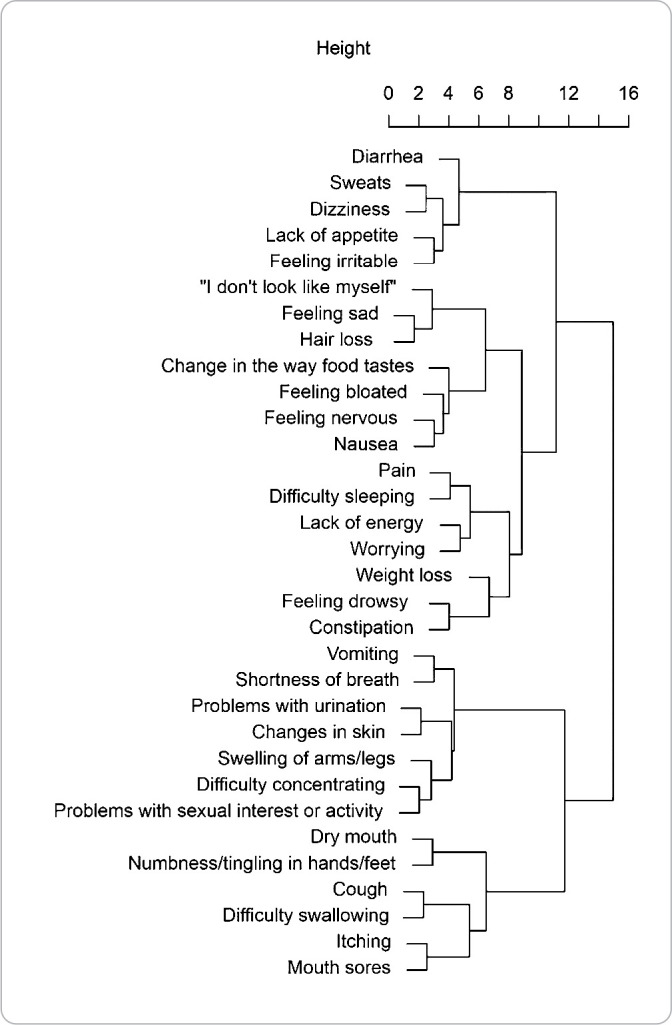
Dendrogram of cancer symptom clusters in hospitalized women with non-metastatic breast cancer, Vitória, Espírito Santo, Brazil, 2023

Figure 1 presents a dendrogram drawn from a symptom cluster reported by 100 hospitalized patients diagnosed with non-metastatic breast cancer as well as the symptoms that occurred together or independently. Hierarchical cluster analysis was used to aggregate self-reported symptoms. Through this representation, it is possible to identify symptoms that are related (connected vertical lines) and the distances between them. The distance values from 0 to 16 refer to the relative distances between symptoms, representing the probability of them being found in the same cluster. From left to right, the data show a close relationship between neuropsychological symptom clusters and gastrointestinal symptom clusters. The nodes of this dendrogram represent the links between symptoms. For instance, a cluster “pain and difficulty sleeping” was more closely associated with the symptoms “lack of energy” and “worrying” than with the cluster “problems with urination and changes in skin”.

## DISCUSSION

This study describes the most prevalent oncological symptoms in hospitalized women with non-metastatic malignant breast cancer. More than 50% of patients in the sample were between 50 and 64 years old, and 51% were in stage I of the disease. Also, 64% of patients had already undergone previous surgery to remove the tumor. The most prevalent oncological symptoms in these patients were, respectively, pain (67%), lack of energy (63%), worrying (62%), and difficulty sleeping (57%), constituting a neuropsychological symptom cluster.

The most prevalent age range for breast cancer presented in this study can be explained by the greater number of screenings and, consequently, diagnoses, through mammography, which the Ministry of Health recommends being performed between the ages of 50 and 69 for asymptomatic women^(21)^, in order to identify breast neoplasia in its preclinical phase^(3,22)^. Furthermore, according to INCA^(22)^, malignant breast neoplasia is rare before the age of 35, with an increase in incidence after the age of 50.

The majority (81%) of women in the sample of this study are from the Metropolitan Health Region of the state of Espírito Santo. The Oncology Care Network of Espírito Santo covers three health regions: Central-North; Metropolitan; and South^(15)^. The Metropolitan Region has a large population concentration (724 inhabitants/km^2^), which represents half of the state’s population, and is concentrated in the municipalities of the Greater Vitória Metropolitan Region (In Portuguese, *Região Metropolitana da Grande Vitória* - RMGV), such as Cariacica, Fundão, Guarapari, Serra, Viana, Vila Velha and Vitória - the state capital. Its boundaries are: to the east, the Atlantic Ocean; to the north, the state of Bahia; to the west and northwest; the state of Minas Gerais; and to the south, the state of Rio de Janeiro^(15)^.

Our results showed that the most frequent histological type in the sample was ductal *in situ* (38%), followed by invasive carcinoma (33%), supporting the literature^(4,23)^. In fact, the most common and most aggressive types of cancer are ductal carcinoma *in situ*, invasive ductal carcinoma, and invasive lobular carcinoma. It is expected that eight out of every ten invasive breast cancers will be ductal carcinoma *in situ* ^(23)^. Once the tumor is detected, breast cancer can be classified as invasive or non-invasive (*in situ*), depending on the site. When the tumor has not spread outside the lobule or ducts, it is called non-invasive, classified as ductal carcinoma *in situ* and lobular carcinoma *in situ* ^(22,23)^.

Ductal carcinoma *in situ* occurs when atypical cells develop within the milk ducts but do not spread into nearby tissue or beyond. The atypical cells can progress and develop into invasive breast cancer^(22,23)^. Lobular carcinoma *in situ* develops in the breast lobules and does not extend outside of these lobules. This carcinoma is considered a precursor to the successive growth of invasive cancer^(22,23)^.

In this study, the highest prevalence of patients with malignant breast neoplasia was in stage I of the disease, and at this stage, the diagnosis is often made in women who are still asymptomatic, who are generally detected in routine tests, without any symptoms, at the age recommended by the Ministry of Health^(21)^. This statement supports the finding that the highest prevalence of breast cancer occurs in women aged between 50 and 69 years and in stage I. In relation to the most prevalent oncological symptoms in these patients, the most prominent were pain (67%), lack of energy (63%), worrying (62%) and difficulty sleeping (57%), constituting a neuropsychological symptom cluster. The symptom pain was the most prevalent, reported by 67% of women with non-metastatic breast cancer, followed by lack of energy (63%), and this finding is in agreement with a study that analyzed the prevalence and comorbidity of pain and lack of energy in women with breast cancer^(24)^, identifying that the occurrence of pain occurred in 47.2% of the sample, and lack of energy, in 51.6%.

Cancer-related lack of energy is a very common symptom, occurring in 63% of patients assessed and with a moderate degree of intensity and discomfort. This lack of energy, represented by tiredness and lack of energy, does not improve with rest or quality sleep, compromising daily activities, such as motor, cognitive and functional coordination of patients. Even simple daily activities, within the home, become exhausting^(24)^. A study that included a sample of 28 women with breast cancer at different stages of the disease found that high concentrations of the IL-1 β receptor antagonist (IL-1βra) were correlated with high levels of lack of energy (p<0.03)^(14)^. Researchers demonstrated an association between polymorphisms in the genes encoding IL-1β and IL-6 and cancer-related lack of energy. Predictors of lack of energy included the presence of at least one cytokine in the IL1β-511 allele (95% CI=0.91-16.6, p=0.007) and homozygosity for any variant of the IL6-174 genotype (G/G or C/C; 95% CI=1.12-17.9, p=0.027)^(25)^.

Evidence supports a strong association between depression, anxiety, cachexia and high levels of expression of cytokines IL-1β, IL-6, IL-10, TNF-α, INF-γ and fractalkine (CX3X) in cancer patients^(26,27)^. These signaling proteins are essential mediators and play a central role in the immune response directed at tumors, since they are intrinsically associated with the process of carcinogenesis in the tumor microenvironment, acting both in signaling tumor growth and in the development of metastases^(28)^. Studies have shown that alterations in genes encoding pro-inflammatory cytokines (IL-1β, IL-6) and their high concentrations contribute greatly to the occurrence, intensity and severity of various symptoms in cancer patients^(14,25)^.

Single nucleotide genetic polymorphisms of IL-1β, IL-10 and TNFR2 have contributed to the identification of patients at high risk for a given set of symptoms. In a study carried out with patients diagnosed with lung cancer, it was observed that, in the 55 polymorphisms analyzed, an additive effect of the mutant alleles of IL-1β, IL-10 and TNFR2 genes were predictive for the cluster of severe pain, depressed mood and lack of energy in these patients. Furthermore, polymorphism in the gene encoding IL-10 was correlated with a significant increase in the risk for lack of energy, i.e., women with the Lys/Glu genotype had a 0.49-fold lower risk of severe lack of energy than those women who exhibited the Lys/Lys genotype (OR = 0.49, 95% CI = 0.25-0.92, p = 0.027)^(29)^.

A study that aimed to explore whether participation in symptom classes of lack of energy, difficulty sleeping, depressed mood and anxiety was associated with other symptoms at moderate to severe levels showed that women with greater difficulty sleeping reported more moderate to severe lack of energy, depressed mood, anxiety and cognitive difficulties^(30)^.

Worrying was a very common symptom in the study, with high prevalence (62%), moderate intensity and discomfort. A study that assessed depressive syndrome, which presents with symptoms of excessive worrying and feeling sad, showed that excessive worrying is also associated with the presence of pain and fear of relapse, sometimes causing lower adherence to therapy^(31)^. A review study showed that anguish, anxiety, psychological distress and depressed emotional function are symptoms that accompany patients from the diagnosis phase to the post-treatment period, being factors that strongly impact the psychological domain^(32)^. Furthermore, patients who present greater frequency, intensity and discomfort of symptoms anguish, anxiety, psychological distress and depressed emotional function have a worse quality of life^(32)^.

It is also important to highlight that hair loss had a prevalence of 43%, with moderate to high frequency, intensity and discomfort in patients, reiterating the impact that hair loss can have on psychological and physical issues for these women^(4)^. Furthermore, symptoms such as nausea, diarrhea, constipation, lack of appetite and weight loss, which directly impact patients’ nutritional status, were also very present, with a prevalence between 30 and 40% of patients.

Other symptoms that occurred with moderate frequency, intensity and discomfort were gastrointestinal symptoms, such as nausea, lack of appetite and vomiting, which directly impact patients’ weight loss. It is worth noting that malnutrition is one of the most frequent disorders in cancer patients, especially when undergoing antineoplastic treatment, exposing them to worse prognoses and clinical complications^(33)^. Due to these symptoms, many patients stop eating and experience loss of appetite or premature satiety, resulting in weight loss, which can progress to a more serious condition: cachexia^(33)^.

A cross-sectional observational study conducted in CACON in southeastern Brazil, which aimed to assess the nutritional status of women undergoing treatment for breast cancer in stages I, II or III, found significant associations between nutritional risk and educational level (p=0.03) and Body Mass Index (BMI) (p=0.01). Binary logistic regression analysis revealed a significant association between educational level and nutritional risk, indicating that lower educational level was associated with higher odds of nutritional risk (OR = 4.59; 95% CI = 1.01-21.04; p=0.049). Furthermore, regarding BMI, it was observed that a BMI above 20.5 kg/m^2^ was associated with a higher probability of nutritional risk (OR = 0.09; 95% CI = 0.01-0.89; p=0.039)^(34)^. However, in another study, it was shown that inflammatory markers, such as the neutrophil-lymphocyte ratio and the platelet-lymphocyte ratio of hospitalized patients with non-metastatic breast cancer, did not show a significant association with nutritional factors^(35)^.

A recent study that aimed to assess and compare the nutritional status of women with breast cancer stages I to III in the first and third cycles of outpatient chemotherapy and to identify associated factors showed that overweight was predominant in both chemotherapy cycles. Approximately 6.67% and 10% of patients presented nutritional risk in the 1^st^ and 3^rd^ cycles of chemotherapy, respectively. Anxiety and depression were prevalent in the 1^st^ cycle of chemotherapy, and were significantly associated with nutritional risk (p=0.002). The variables age in the 3^rd^ cycle and pain/discomfort in the 1^st^ cycle (p=0.049 and p=0.043, respectively) presented a significant association with nutritional risk^(36)^.

### Study limitations

The study has some limitations. The data were collected from a single oncology referral center in Brazil, with a small sample size. Furthermore, the descriptive observational design does not allow for bivariate and multivariate associations. Another limitation was the inclusion of patients with non-metastatic breast cancer in stages I, II and III, with different chemotherapy protocols and in any phase of antineoplastic treatment.

### Contributions to Nursing

A better understanding of how the pattern of cancer symptom clusters is configured in hospitalized women with non-metastatic breast cancer is crucial, in order to enable the development of personalized interventions that can contribute to better care for these patients, with a positive impact on their quality of life^(37-40)^. The work of multidisciplinary and interprofessional teams is necessary to manage the cancer symptom clusters presented by these patients, through personalized care focused on each patient’s preferences.

## CONCLUSIONS

Neuropsychological symptom cluster 3 (pain, lack of energy, worrying and difficulty sleeping) was the most prevalent in women hospitalized with nonmetastatic breast cancer.
