# Supplementary material for: Cancer symptom cluster in hospitalized women with breast cancer: a descriptive observational study
Source: Rev Bras Enferm. 2025 Apr 28;78(2):e20240091. doi: 10.1590/0034-7167-2024-0091 (PMC12037190; doi:10.1590/0034-7167-2024-0091)
Supplement: Translation

# Translation: Cluster de sintomas oncológicos em mulheres com neoplasia maligna de mama não metastático hospitalizadas

**DOI:** 10.1590/0034-7167-2024-0091pt

**Authors:** Roberto Júnio Gomes Silva, Wesley Rocha Grippa, Raphael Manhães Pessanha, Júlia Anhoque Cavalcanti Marcarini, Luiz Cláudio Barreto Silva, Naira Santos D’Agostini, Luís Carlos Lopes-Júnior

**Affiliations:** I Universidade Federal do Espírito Santo. Vitoria, Espírito Santo, Brasil

**Keywords:** Sinais e Sintomas, Sintomas Concomitantes, Neoplasias da Mama, Oncologia, Enfermagem Oncológica

## Abstract

**Objetivos:**

descrever e analisar os sintomas oncológicos mais prevalentes, bem como sua intensidade, incômodo e clusterização em mulheres com neoplasia maligna da mama não metastático hospitalizadas.

**Métodos:**

estudo observacional descritivo, com 100 mulheres hospitalizadas com câncer de mama não metastático, recrutadas em centro de referência em oncologia do sudeste brasileiro entre junho de 2022 e março de 2023. Para análise dos *clusters* de sintomas oncológicos, foi utilizado o método hierárquico.

**Resultados:**

51 pacientes se encontravam em estágio I da doença, 13 mulheres, em estágio II, 36 mulheres, em estágio III. O tipo histológico mais frequente foi carcinoma *ductal in situ* (38%), seguido do carcinoma invasivo (33%). Os sintomas oncológicos mais prevalentes foram dor (67%), fadiga (63%), preocupações (62%), distúrbios do sono (57%), constituindo *cluster* de sintoma neuropsicológico.

**Conclusões:**

o *cluster* de sintoma neuropsicológico (dor-fadiga-preocupações-distúrbio do sono) foi o mais prevalente em mulheres hospitalizadas com neoplasia maligna da mama não metastático.

## INTRODUÇÃO

A neoplasia maligna da mama é considerada o tumor mais incidente e com maior mortalidade no sexo feminino não só no Brasil, mas também em todo o mundo^(1,2)^. De acordo com dados do Instituto Nacional do Câncer (INCA), esse tipo de neoplasia é responsável por 28% dos casos novos anuais de todos os tipos de câncer^(3)^. Estima-se que, no Brasil, uma em cada 12 mulheres desenvolverá essa patologia ao longo da vida^(3)^.

Pacientes com neoplasia maligna da mama experienciam uma série de sintomas ocasionados pelo próprio microambiente tumoral e também associados aos efeitos colaterais do tratamento antineoplásico utilizado^(4-6)^. Esse *cluster* de sintomas que se inter-relacionam é chamado de *cluster* de sintomas oncológicos, os quais podem ser definidos como dois ou mais sintomas relacionados entre si e que ocorrem em conjunto, sem obrigatoriamente se relacionarem com outros *clusters*, sendo a relação entre os sintomas do mesmo *cluster* mais forte do que a relação com diferentes *clusters* ^(7-10)^.

Os sintomas experimentados por pacientes com câncer de mama podem ser de origem física, mental e psicológica, e incluem fadiga relacionada ao câncer, dor, distúrbios do sono, ansiedade, depressão e função cognitiva diminuída^(7,8,11)^, os quais geralmente estão associados aos tratamentos antineoplásicos ou ao próprio tumor^(7,8)^. Outros sintomas muito comuns são náusea, vômito, inapetência, diarreia e/ou constipação, boca seca e falta de apetite, os quais impactam diretamente o estado nutricional dos pacientes, gerando déficit no consumo alimentar, com consequente perda de peso e de massa muscular^(7,8,11)^.

Os primeiros estudos envolvendo *cluster* de sintomas oncológicos são oriundos de experimentações com murinos submetidos à indução de quadros infecciosos e à injeção de citocinas pró-inflamatórias, desencadeando quadros inflamatórios nesses animais, o que culminou no fenômeno de “comportamento doentio” (do inglês, *sickness behavior*)^(6,7)^. O comportamento doentio foi estabelecido em 1992 para designar um conjunto de alterações comportamentais que cursavam com diversificados processos patológicos e inflamatórios, como a perda aparente do interesse desses animais por atividades cotidianas, como busca por alimentos, diminuição do interesse sexual e da interação social^(6,7,12)^.

Estudos em animais e em humanos têm mostrado que a infusão de citocinas também induz o fenômeno de comportamento doentio^(6-8,12)^. De fato, similarmente ao fenômeno do comportamento doentio, apresentado por animais de laboratório, em humanos, especialmente em pacientes com câncer, foram observados quadros de dor oncológica, fadiga relacionada ao câncer, distúrbios do sono, ansiedade e depressão associados aos altos níveis de expressão de citocinas pró-inflamatórias expressas^(6-8,11,12)^, evidenciando a estreita relação entre o padrão de comportamento doentio visto nos experimentos com animais e o padrão de *clusters* de sintomas oncológicos relatados por pacientes com câncer^(8,12)^.

Evidências científicas suportam de forma consistente que mecanismos biológicos comuns podem estar subjacentes à interação entre os sistemas nervoso, endócrino e imune, os quais orquestram um conjunto de respostas capazes de instalar mudanças comportamentais e fisiológicas tanto no organismo animal quanto no organismo humano^(6-8,11,12)^. Ademais, estudos que abarcam o comportamento doentio em experimentação animal, bem como os *clusters* de sintomas oncológicos em humanos, suportam a hipótese de que citocinas pró-inflamatórias são um dos mecanismos biológicos subjacentes ao surgimento desses *clusters* sintomatológicos, especialmente a liberação de IL-1β, IL-6, IL-8, TNF-α, IL-12p70 e IFN-y^(6-8,11)^. Esse padrão de expressão de citocinas pró-inflamatórias resulta, por exemplo, nos *clusters* de sintomas oncológicos em pacientes com câncer, compostos pelos sintomas fadiga relacionada ao câncer, humor deprimido, depressão, distúrbios do sono e aumento da sensibilidade à dor^(6-8,11,12)^.

No microambiente tumoral, citocinas estimulam a resposta do hospedeiro para controlar o estresse celular e reduzir o dano celular^(4,6,11)^. Assim, alterações em citocinas e em outros processos neuroimunológicos podem ser críticas para a produção de *clusters* de sintomas oncológicos em pacientes com câncer e, potencialmente, podem ser consideradas como alvo para prevenção e tratamento desses sintomas^(6-8,10-13)^. As citocinas IL-1, IL-6 e TNF-α estão implicadas, por exemplo, na fisiopatologia da fadiga relacionada ao câncer e a distúrbios do sono^(6,11)^. Estudo conduzido com mulheres com câncer de mama, em diferentes estágios da doença, evidenciou que altas concentrações do antagonista do receptor de IL-1β (IL-1βra) estiveram associadas a altos níveis de fadiga relacionada ao câncer^(14)^.

Os inúmeros e complexos sintomas oncológicos que podem ser apresentados pelas pacientes com câncer de mama impactam negativamente sua qualidade de vida relacionada à saúde^(4)^. Ademais, estes sintomas estão associados a piores prognósticos, incluindo baixa sobrevida e baixa adesão ao tratamento oncológico^(4,12)^. A importância de se avaliar e intervir sob tais *clusters* de sintomas, não só em termos de sobrevida do paciente, mas, sobretudo, em termos de qualidade de vida durante e após o tratamento, está bem estabelecida e é atualmente parte integrante dos pilares da pesquisa em oncologia e, particularmente, em enfermagem oncológica^(12,13)^. A descrição e a análise desses sintomas e sua clusterização se tornam cruciais para nortear a atuação da equipe multidisciplinar e interprofissional na abordagem para um cuidado personalizado a essas pacientes.

## OBJETIVOS

Descrever e analisar os sintomas oncológicos mais prevalentes, bem como sua intensidade, incômodo e clusterização em mulheres com neoplasia maligna da mama não metastático hospitalizadas.

## MÉTODOS

### Aspectos éticos

O estudo foi aprovado pelo Comitê de Ética em Pesquisa do Centro de Ciências da Saúde da Universidade Federal do Espírito Santo, em 10 de maio de 2022, sob Certificado de Apresentação para Apreciação Ética (CAAE) e Parecer de aprovação, em consonância com as diretrizes e normas regulamentadoras de pesquisa envolvendo seres humanos no Brasil, estabelecidas pela Resolução nº 466/2012. Salienta-se que todos os pacientes foram abordados e informados a respeito do conteúdo e objetivos da pesquisa, manifestando sua concordância em participar voluntariamente do estudo mediante a assinatura do Termo de Consentimento Livre e Esclarecido antes do início da pesquisa, em duas vias, sendo que uma foi entregue ao participante e a outra ficou em posse do pesquisador principal. Ressalta-se que foram respeitados o anonimato e a privacidade das participantes de pesquisa.

### Desenho, período e local do estudo

Estudo observacional descritivo com abordagem quantitativa, norteado pelas recomendações do *STrengthening the Reporting of OBservational studies in Epidemiology* (STROBE).

O estudo foi conduzido no Setor de Internação A do Afecc-Hospital Santa Rita de Cássia (HSRC), localizado na capital Vitória, Espírito Santo, Brasil. O HSRC é uma entidade filantrópica, criado em 1970, único Centro de Assistência de Alta Complexidade em Oncologia (CACON) do estado e reconhecido em todo o Espírito Santo como referência em tratamento para câncer. O hospital mantém caráter filantrópico, e destina 60% de seus atendimentos a pacientes do Sistema Único de Saúde. Por isso, recebe também pacientes de outros estados, como sul da Bahia, leste de Minas Gerais e norte do Rio de Janeiro. Possui um Registro Hospitalar de Câncer (RHC) estruturado que funciona desde 2000, com seus bancos de dados encaminhados anualmente ao Sistema Integrado do Registro Hospitalar de Câncer (SISRHC)^(15)^.

### População, critérios de inclusão e exclusão

Com base na última estimativa do INCA do Brasil para o triênio 2023-2025, a incidência de câncer de mama feminino é de 74 mil casos para o Brasil, sendo 84,46/100 mil casos esperados para a região Sudeste^(3)^. A casuística do serviço no Setor de Internação A do HSRC (local da coleta de dados) foi de 225 casos internados com diagnóstico de neoplasia maligna da mama em diferentes fases do tratamento antineoplásico, no ano de 2019, e 182 casos, em 2020, configurando uma média de casos no biênio de 204 casos/ano. Consideramos no nosso cálculo apenas prevalência dos casos de neoplasias malignas da mama no ano de 2019, visando diminuir o viés da pandemia do novo coronavírus, que alterou sobremaneira o fluxo de pacientes e de internações no ano de 2020.

A partir da fórmula utilizada para o cálculo amostral, qual seja n=N.Z^2^. p.(1-p) / Z^2^.p.(1-p) + e^2^.N-1, em que n significa amostra calculada, N, população, Z, variável normal, p, real probabilidade do evento, e erro amostral^(16)^, e considerando a população de pacientes diagnosticados com câncer de mama no HSRC em 2019 (n= 225), tendo como base a Classificação Internacional de Doenças (CID-10) C50 (Neoplasia maligna da mama) e fixando α em 5% (erro amostral), com nível de confiança de 95% e um β de 0,2 (conferindo 80% de poder de teste), com um percentual mínimo de 12%, obteve-se o n amostral dessa pesquisa igual a 95 pacientes.

Foram incluídas neste estudo pacientes do sexo feminino, com idade acima de 18 anos, diagnóstico anatomopatológico de câncer de mama em estágios I, II ou III, tendo como base a CID-10 C50 (Neoplasia maligna da mama), em qualquer fase do tratamento antineoplásico.

Foram excluídas pacientes em estágio IV, em cuidado paliativo exclusivo, que internaram para cirurgia reparadora ou por intercorrências clínicas não relacionadas ao câncer de mama. Salienta-se que não houve perdas e nem recusas de pacientes no presente estudo.

### Protocolo do estudo

A coleta de dados ocorreu entre junho de 2022 e março de 2023. As participantes da pesquisa que atenderam aos critérios de elegibilidade foram recrutadas a partir da admissão no Setor de Internação A do HSRC. Um questionário sociodemográfico e clínico foi elaborado pelos pesquisadores, o qual foi estruturado em três componentes, a saber: I) anamnese (história da doença oncológica, história pregressa e riscos cardiovasculares, história familiar, hábitos de vida, nutrição, prática atividade física); II) exame físico geral e específico composto de sinais vitais, exame da cabeça/pescoço/neurológico, tórax/pulmões/coração e abdome; III) exames laboratoriais que faziam parte da rotina do hospital e foram acessados do próprio prontuário das pacientes (hemograma e glicemia capilar). As variáveis sociodemográficas incluídas no questionário foram idade, cor, procedência, renda, escolaridade, ocupação, estado civil, número de filhos, consumo de tabaco e de bebida alcóolica. As variáveis clínicas que compuseram o questionário foram classificação diagnóstica, tipo histológico, tumor-nódulo-metástase, estágio, doenças pregressas e cirurgias prévias. Ressalta-se que todos os dados foram coletados via aplicação de questionário à beira do leito por um enfermeiro oncologista (primeiro autor desta pesquisa). Ademais, salientamos que foi obtida a permissão da direção do hospital e do Setor de Internação A do HSRC sobre a coleta de dados do prontuário das pacientes.

Para avaliação dos sintomas oncológicos, foi utilizado o *Memorial Symptom Assessment Scale* (MSAS)^(17)^. O MSAS foi desenvolvido por Portenoy e colaboradores (1994)^(18)^. O MSAS é um instrumento que auxilia na detecção e monitoramento de múltiplos sintomas em pacientes com câncer^(18)^, e é utilizado mundialmente na área da oncologia. O MSAS traz a combinação de diferentes sintomas, com seus respectivos graus de frequência, intensidade e incômodo. Trata-se de instrumento de autorrelato no qual os pacientes atribuem um valor numérico de 1 a 4 pontos para a frequência e intensidade de 32 sintomas e de 0 a 4 para o grau de incômodo experimentado durante a última semana. Ainda, é dividido em subescalas, que avaliam sintomas psicológicos (PSYCH), com seis itens, e sintomas físicos (PHYS H e PHYS L), com 26 itens^(18)^.

Utilizamos a versão validada para o português do Brasil, que apontou que a confiabilidade da escala se mostrou satisfatória nos testes-retestes^(19)^. Os valores de Kappa obtidos para cada item da escala apresentaram-se adequados, sendo o maior item de 0,96 e o menor de 0,69. Também avaliou-se o Kappa das subescalas, sendo de 0,84 para sintomas físicos de alta frequência, de 0,81, para sintomas físicos de baixa frequência, de 0,81, também para sintomas psicológicos, e de 0,78, para Índice Geral de Sofrimento. Os autores concluíram que os altos níveis de confiabilidade estimados permitem afirmar que o processo de aferição dos itens do MSAS foi adequado^(19)^.

### Análise dos resultados e estatística

Os dados foram tabulados em planilha da *Microsoft Excel* ^®^ e analisados por meio do *software Statistical Package for the Social Sciences* (SPSS), versão 28.0. Os resultados do estudo foram apresentados em estatística descritiva, contendo frequências absolutas e porcentagens para todas as variáveis estudadas. Já as variáveis idade e aquelas referentes ao MSAS foram apresentadas por meio da média e desvio padrão. Para análise de *cluster* de sintomas, foi utilizado o método hierárquico^(20)^. A análise de *cluster* pelo método hierárquico é uma técnica estatística exploratória que agrupa observações em *clusters* ou grupos com base em suas similaridades ou dissimilaridades. O resultado desse *cluster* foi apresentado na forma de dendrograma, que consiste na representação visual desses *clusters*, em que os grupos mais semelhantes são unidos primeiro e os mais diferentes estão localizados em extremidades opostas do dendrograma. O método hierárquico minimiza a variância total dentro dos *clusters*, e é frequentemente usado em análise de *clusters* ^(20)^. Foi utilizada a distância euclidiana como medida de similaridade entre os sintomas na hierarquização, buscando agrupá-los de acordo com sua proximidade em um espaço multidimensional baseado nos atributos de frequência, intensidade e incômodo. Esta abordagem permitiu a identificação dos grupos de sintomas relacionados entre si, proporcionando uma compreensão hierárquica das relações entre os sintomas do MSAS. O nível de significância utilizado em todas as análises foi de 5%.

## RESULTADOS

Participaram do estudo 100 mulheres com diagnóstico de câncer de mama não metastático hospitalizadas. As características demográficas e clínicas da amostra estão apresentadas na Tabela 1 e Tabela 2, respectivamente. A média de idade das participantes foi de 59,15 anos e desvio padrão de 10,27 anos de idade. Metade da amostra era representada por mulheres pardas (50%), casadas (50%), com dois ou mais filhos (59%), tendo como o nível de escolaridade o ensino fundamental completo (44%). A maioria residia na Região Metropolitana do estado do Espírito Santo (81%), mulheres do lar (43%), com renda de um salário mínimo (70%), não tabagistas (78%) e não etilistas (86%) (Tabela 1).

**Tabela 1:** Caracterização sociodemográfica e de hábitos/estilo de vida de mulheres com câncer de mama não metastático hospitalizadas, Vitória, Espírito Santo, Brasil, 2023

Variáveis	*n*	%
Idade (em anos)		
Média (desvio padrão)	59,15 (10,27)	-
Faixa etária		
< 50 anos	15	15,00
50-64 anos	53	53,00
≥ 65 anos	32	32,00
Cor autorreferida		
Branca Negra	38 12	38,00 12,00
Parda	50	50,00
Região de saúde		
Região Metropolitana	81	81,00
Região Central-Norte	14	14,00
Região Sul	5	5,00
Fonte de renda		
Aposentado	56	56,00
Pensionista	9	9,00
Empregado	17	17,00
Sem renda	18	18,00
Renda		
< 1 salário mínimo	7	7,00
1 salário mínimo	70	70,00
2 salários mínimos	16	16,00
≥ 3 salários mínimos	7	7,00
Escolaridade		
Analfabeto	9	9,00
Fundamental	45	45,00
Médio	32	32,00
Superior	14	14,00
Ocupação		
Do lar	43	43,00
Doméstica	22	22,00
Outra^*^	35	35,00
Estado civil		
Solteiro	21	21,00
Casado	50	50,00
Viúvo	15	15,00
Divorciado	14	14,00
Filhos		
Nenhum	16	16,00
1	25	25,00
≥ 2	59	59,00
Faz uso de tabaco?		
Não	93	93,00
Sim	7	7,00
Já fumou?		
Não	78	78,00
Sim	22	22,00
Faz uso de bebida alcóolica?		
Não	86	86,00
Sim	14	14,00

* *Outra: cabeleireira, costureira, professora, guia de turismo, cozinheira.*

**Tabela 2:** Perfil clínico de mulheres com câncer de mama não metastático hospitalizadas, Vitória, Espírito Santo, Brasil, 2023

Variáveis	*n*	%
Diagnóstico de câncer atual		
Neoplasia maligna da mama	37	37,00
Neoplasia maligna da mama central	20	20,00
Neoplasia maligna do quadrante superior interno da mama	19	19,00
Neoplasia maligna do quadrante inferior interno da mama	2	2,00
Neoplasia maligna do quadrante superior externo da mama	2	2,00
Neoplasia maligna do quadrante inferior externo da mama	6	6,00
Neoplasia maligna da porção axilar da mama	7	7,00
Neoplasia maligna da mama com lesão invasiva	7	7,00
Tipo histológico		
Carcinoma invasivo	33	33,00
Carcinoma ductal *in situ*	38	38,00
Carcinoma lobular *in situ*	29	29,00
Tumor-nódulo-metástase		
T1N1M0	8	8,00
T1N2M0	13	13,00
T2N1M0	14	14,00
T2N2M0	29	29,00
T3N1M0	21	21,00
T3N2M0	6	6,00
T4N2M0	5	5,00
T4N3M0	4	4,00
Estágio		
I	51	51,00
II	13	13,00
III	36	36,00
Hipertensão arterial sistêmica		
Não	71	71,00
Sim	29	29,00
Dislipidemia		
Não	96	96,00
Sim	4	4,00
Diabetes Mellitus		
Não	87	87,00
Sim	13	13,00
Cirurgia prévia		
Não	36	36,00
Sim	64	64,00

Entre as 100 mulheres com câncer de mama não metastático, 51 pacientes se encontravam em estágio I da doença, 13 mulheres, em estágio II, e 36 mulheres, em estágio III. O tipo histológico mais frequente foi o carcinoma ductal *in situ* (38%), seguido do carcinoma invasivo (33%). Entre as participantes da pesquisa, 29% das mulheres que compuseram a amostra eram hipertensas; 13% tinham diabetes; e 64% já tinham passado por alguma cirurgia. Tais dados estão detalhados na Tabela 2.

A Tabela 3 apresenta a frequência, a intensidade e o incômodo dos sintomas oncológicos de mulheres hospitalizadas com câncer de mama não metastático. Os sintomas mais prevalentes nessas pacientes foram, respectivamente, dor (67%), fadiga (63%), preocupações (62%) e distúrbios do sono (57%), avaliados por meio do MSAS.

**Tabela 3:** Sintomas oncológicos de mulheres com câncer de mama não metastático hospitalizadas segundo frequência, intensidade e incômodo, Vitória, Espírito Santo, Brasil, 2023

Sintomas do MSAS		Prevalência		Frequência	Intensidade	Incômodo
		*n*	%	M ± DP	M ± DP	M ± DP
1	Dificuldade para se concentrar	27	27,00	2,59 ± 0,69	2,30 ± 0,54	3,11 ± 1,48
2	Dor	67	67,00	2,94 ± 0,87	3,09 ± 0,95	3,43 ± 0,97
3	Fadiga	63	63,00	2,97 ± 0,76	2,78 ± 1,04	2,98 ± 1,20
4	Tosse	32	32,00	3,19 ± 0,82	2,25 ± 0,88	2,66 ± 1,36
5	Nervosismo	48	48,00	2,73 ± 0,89	2,60 ± 0,84	3,29 ± 1,07
6	Boca seca	51	51,00	2,86 ± 0,98	2,53 ± 1,05	2,55 ± 1,23
7	Náusea	37	37,00	2,68 ± 0,67	2,59 ± 0,72	3,08 ± 1,19
8	Sonolência	39	39,00	3,18 ± 0,56	2,79 ± 0,83	3,13 ± 1,38
9	Dormência ou formigamento nas mãos	40	40,00	2,63 ± 1,00	2,70 ± 1,26	2,80 ± 1,20
10	Distúrbios do sono	57	57,00	3,18 ± 0,68	3,19 ± 0,77	3,23 ± 0,96
11	Empanzinado	40	40,00	3,05 ± 0,64	2,83 ± 0,68	3,03 ± 0,99
12	Problemas para urinar	1	1,00	2,00	2,00	1,00
13	Vômito	18	18,00	2,11 ± 0,58	2,33 ± 0,77	3,00 ± 0,84
14	Falta de ar	26	26,00	2,88 ± 0,71	3,00 ± 0,69	2,77 ± 1,07
15	Diarreia	36	36,00	2,39 ± 0,96	2,03 ± 0,51	2,86 ± 0,93
16	Tristeza	38	38,00	3,32 ± 0,87	3,32 ± 0,90	3,50 ± 0,83
17	Suor	42	42,00	2,38 ± 0,54	2,38 ± 0,66	2,93 ± 0,95
18	Preocupações	62	62,00	3,16 ± 0,85	2,82 ± 0,98	2,85 ± 1,51
19	Problemas com desejo/atividade sexual	18	18,00	3,06 ± 0,64	2,44 ± 0,51	2,83 ± 1,76
20	Coceira	17	17,00	1,88 ± 0,60	1,82 ± 0,64	1,71 ± 1,31
21	Falta de apetite	43	43,00	2,91 ± 0,81	2,58 ± 0,73	2,33 ± 1,21
22	Tontura	32	32,00	2,41 ± 0,50	2,22 ± 0,71	2,81 ± 1,18
23	Dificuldade para engolir	26	26,00	2,85 ± 0,73	2,38 ± 0,80	2,69 ± 1,38
24	Irritado	36	36,00	2,69 ± 0,52	2,64 ± 0,59	2,83 ± 0,91
25	Feridas na boca	15	15,00	1,80 ± 0,77	1,80 ± 0,94	2,13 ± 1,30
26	Mudança no gosto dos alimentos	37	37,00	3,14 ± 0,89	2,68 ± 0,78	2,76 ± 1,36
27	Perda de peso	42	42,00	2,81 ± 0,94	2,95 ± 1,01	2,40 ± 1,71
28	Alopecia	43	43,00	3,35 ± 0,90	3,16 ± 0,90	3,51 ± 1,01
29	Constipação	43	43,00	2,67 ± 0,78	2,60 ± 0,90	2,53 ± 1,42
30	Inchaço nos braços ou pernas	19	19,00	3,05 ± 0,85	2,79 ± 1,18	2,68 ± 1,60
31	“Eu não pareço mais a mesma”	31	31,00	3,10 ± 0,98	2,90 ± 1,04	3,26 ± 1,12
32	Alterações na pele	12	12,00	2,50 ± 0,67	2,42 ± 0,79	1,75 ± 0,87

*MSAS - Memorial Symptom Assessment Scale (Intervalo de Confiança de 95%); M - média; DP - desvio padrão.*

O Quadro 1 apresenta a hierarquização dos *clusters* obtidos por meio do MSAS segundo os pressupostos do método de *cluster* hierárquico, conforme descrito na seção método.

**Quadro 1:** Identificação dos *clusters* de sintomas oncológicos a partir do *Memorial Symptom Assessment Scale* de mulheres com câncer de mama não metastático hospitalizadas segundo método de Ward, Vitória, Espírito Santo, Brasil, 2023

*Cluster*	Sintomas do MSAS
**1**	Diarreia
	Suor
	Tontura
	Falta de apetite
	Irritado
**2**	“Eu não pareço mais a mesma”
	Tristeza
	Alopecia
	Mudança no gosto dos alimentos
	Empanzinado
	Nervosismo
	Náusea
**3**	Dor
	Distúrbios do sono
	Fadiga
	Preocupações
**4**	Perda de peso
	Sonolência
	Constipação
**5**	Vômito
	Falta de ar
	Problemas para urinar
	Alterações na pele
	Inchaço nos braços ou pernas
	Dificuldade para se concentrar
	Problemas com desejo/atividade sexual
**6**	Boca seca
	Dormência ou formigamento nas mãos
	Tosse
	Dificuldade para engolir
	Coceira
	Feridas na boca

*MSAS - Memorial Symptom Assessment Scale.*

O *cluster* de sintoma neuropsicológico 3 (dor, fadiga, preocupações e distúrbio do sono) foi o mais prevalente na amostra.

A Figura 1 apresenta um dendrograma elaborado a partir do *cluster* de sintomas reportados pelas 100 pacientes diagnosticado com câncer de mama não metastático hospitalizadas, bem como dos sintomas que ocorreram em conjunto ou independentemente. Foi utilizada a análise de *cluster* hierárquico para agregar os sintomas autorreferidos. Por meio dessa representação, é possível identificar os sintomas que estão relacionados (linhas verticais conectadas) e as distâncias entre os mesmos. Os valores de distância de 0 a 16 referem-se às distâncias relativas entre os sintomas, representando a probabilidade de serem encontrados em um mesmo *cluster.* Da esquerda para a direita, os dados mostram uma estreita relação entre os *clusters* de sintomas neuropsicológicos e os *clusters* de sintomas gastrointestinais. Os nós desse dendrograma representam as ligações entre os sintomas. Por exemplo, um *cluster* “dor e distúrbios do sono” esteve associado de forma mais próxima aos sintomas “fadiga” e “preocupações” do que ao *cluster* “problemas para urinar e alterações na pele”.

**Figura 1:** Dendrograma dos *clusters* de sintomas oncológicos em mulheres com câncer de mama não metastático hospitalizadas, Vitória, Espírito Santo, Brasil, 2023

## DISCUSSÃO

Este estudo descreve os sintomas oncológicos mais prevalentes em mulheres com neoplasia maligna da mama não metastático e hospitalizadas. Mais de 50% das pacientes da amostra possuíam idade entre 50 e 64 anos e 51% estavam no estágio I da doença, sendo que 64% das pacientes já haviam sido submetidas à cirurgia prévia de retirada do tumor. Os sintomas oncológicos mais prevalentes nessas pacientes foram, respectivamente, dor (67%), fadiga (63%), preocupações (62%), e distúrbios do sono (57%), constituindo um *cluster* de sintoma neuropsicológico.

A faixa etária mais prevalente de câncer de mama apresentada neste estudo pode ser explicada pelo maior número de rastreamento e, consequentemente, de diagnósticos, através da mamografia, cuja recomendação do Ministério da Saúde é a realização entre os 50 e 69 anos para mulheres assintomáticas^(21)^, a fim de identificar a neoplasia mamária em sua fase pré-clínica^(3,22)^. Além disso, de acordo com o INCA^(22)^, a neoplasia maligna da mama é rara antes dos 35 anos de idade, tendo um aumento de sua incidência após os 50 anos.

A maioria (81%) das mulheres da amostra deste estudo é da Região Metropolitana de saúde do estado do Espírito Santo. A Rede de Atenção à Oncologia do Espírito Santo abrange três regiões de saúde: Central-Norte; Metropolitana; e Sul^(15)^. A Região Metropolitana apresenta uma grande concentração da população (724 hab./km^2^), que representa metade da população do estado, e está concentrada nos municípios da Região Metropolitana da Grande Vitória (RMGV), como Cariacica, Fundão, Guarapari, Serra, Viana, Vila Velha e Vitória - capital do estado. Seus limites são: a leste, o Oceano Atlântico; ao norte, o estado da Bahia; a oeste e noroeste; o estado de Minas Gerais; e ao sul, o estado do Rio de Janeiro^(15)^.

Nossos resultados apontaram que o tipo histológico mais frequente na amostra foi o ductal *in situ* (38%), seguido do carcinoma invasivo (33%), corroborando a literatura^(4,23)^. De fato, os tipos de cânceres mais comuns e mais agressivos são o carcinoma ductal *in situ*, o carcinoma ductal invasivo e o carcinoma lobular invasivo. Espera-se que, em cada dez cânceres de mama invasivos, oito sejam carcinomas ductais *in situ* ^(23)^. Após detectado o tumor, o câncer de mama pode ser classificado em invasivo e não invasivo (*in situ*), conforme o sítio. Quando o tumor não se estendeu para fora do lóbulo ou ductos, denomina-se não invasivo, classificados como carcinoma ductal *in situ* e carcinoma lobular *in situ* ^(22,23)^.

O carcinoma ductal *in situ* manifesta-se quando as células atípicas se desenvolvem dentro dos ductos lácteos, mas não se estendem para a proximidade do tecido ou para fora. As células atípicas podem progredir e se transformar em câncer de mama invasivo^(22,23)^. O carcinoma lobular *in situ* desenvolve-se nos lóbulos mamários, não se estendendo para fora desses lóbulos. Esse carcinoma é considerado um antecessor para o crescimento sucessivo de câncer invasivo^(22,23)^.

Nesta pesquisa, a maior prevalência de pacientes com neoplasia maligna da mama se apresentou no estágio I da doença, e nessa fase, muitas vezes o diagnóstico é feito em mulheres ainda assintomáticas, as quais são geralmente detectadas em exames de rotina, sem quaisquer sintomas, na idade recomendada pelo Ministério da Saúde^(21)^. Essa afirmação corrobora o achado de a maior prevalência do câncer de mama ocorrer com mulheres em idade entre 50 e 69 anos e em estágio I. Em relação aos sintomas oncológicos mais prevalentes nessas pacientes, destacaram-se dor (67%), fadiga (63%), preocupações (62%) e distúrbios do sono (57%), constituindo um *cluster* de sintoma neuropsicológico. O sintoma dor foi o mais prevalente, relatado por 67% das mulheres com câncer de mama não metastático, seguido do sintoma fadiga (63%), e este achado está de acordo com estudo que analisou a prevalência e a comorbidade de dor e fadiga em mulheres com câncer de mama^(24)^, identificando que a ocorrência de dor ocorreu em 47,2% da amostra, e a fadiga, em 51,6%.

A fadiga relacionada ao câncer é um sintoma muito comum, apresentando-se em 63% das pacientes avaliadas e com moderado grau de intensidade e incômodo. Essa fadiga, representada pelo cansaço e falta de energia, não melhora com o repouso ou um sono de qualidade, comprometendo as atividades do cotidiano, como coordenação motora, cognitiva e funcional das pacientes. Até mesmo atividades simples do dia a dia, dentro de casa, tornam-se exaustivas^(24)^. Estudo que compreendeu uma amostra de 28 mulheres com câncer de mama em diferentes estágios da doença detectou que altas concentrações do antagonista do receptor de IL-1 β (IL-1βra) estavam correlacionadas com altos níveis de fadiga (p<0,03)^(14)^. Pesquisadores demonstraram uma associação entre polimorfismos dos genes que codificam a IL-1β e IL-6 e a fadiga relacionada ao câncer. Os preditores de fadiga incluíram a presença de pelo menos uma citocina no alelo IL1β-511 (IC95%= 0,91-16,6, p=0,007) e homozigosidade para qualquer variante do genótipo IL6-174 (G/G ou C/C; IC95%=1,12-17,9, p =0,027)^(25)^.

Evidências suportam uma forte associação entre depressão, ansiedade, caquexia e altos níveis de expressão das citocinas IL-1β, IL-6, IL-10, TNF-α, INF-γ e fractalcina (CX3X) em pacientes com câncer^(26,27)^. Essas proteínas de sinalização são mediadoras essenciais, e possuem papel central na resposta imune dirigida a tumores, uma vez que estão intrinsecamente associadas ao processo de carcinogênese no microambiente tumoral, atuando tanto na sinalização do crescimento tumoral quanto no desenvolvimento de metástases^(28)^. Estudos têm demostrado que alterações nos genes codificadores de citocinas pró-inflamatórias (IL-1β, IL-6) e suas concentrações elevadas contribuem sobremaneira para a ocorrência, intensidade e severidade de diversos sintomas em pacientes oncológicos^(14,25)^.

Polimorfismos genéticos de nucleotídeo único da IL-1β, IL-10 e TNFR2 têm contribuído para a identificação de pacientes em risco elevado para um dado conjunto de sintomas. Em estudo realizado com pacientes diagnosticados com câncer de pulmão, observou-se que, nos 55 polimorfismos analisados, um efeito aditivo dos alelos mutantes da IL-1β, IL-10 e os genes TNFR2 foram preditivos para o *cluster* de dor severa, humor deprimido e fadiga nesses pacientes. Além disso, polimorfismo no gene codificante de IL-10 foi correlacionado a um aumento significativo do risco para fadiga, isto é, mulheres com o genótipo Lis/Glu tinham 0,49 vezes menos risco de fadiga severa do que aquelas mulheres que exibiam o genótipo Lis/Lis (OR = 0.49, IC95% = 0.25-0.92, p = 0,027)^(29)^.

Estudo que teve como objetivo explorar se a participação em classes de sintomas de fadiga, distúrbio do sono, humor deprimido e ansiedade estava associada a outros sintomas em níveis moderados a graves mostrou que mulheres com distúrbios do sono de maior gravidade relataram mais fadiga moderada a grave, humor deprimido, ansiedade e dificuldades cognitivas^(30)^.

Sintoma muito presente no estudo, com prevalência elevada (62%), e moderada intensidade e incômodo foi a preocupação. Estudo que avaliou a síndrome depressiva, a qual cursa com sintomas de excesso de preocupação e tristeza, evidenciou que o excesso de preocupação está associado também à presença de dor e medo de recidiva, ocasionando, por vezes, uma menor adesão à terapêutica^(31)^. Estudo de revisão mostrou que angústia, ansiedade, sofrimento psicológico e função emocional deprimida são sintomas que acompanham as pacientes desde a fase do diagnóstico até o período pós-tratamento, sendo fatores que impactam fortemente o domínio psicológico^(32)^. Ademais, pacientes que apresentam maior frequência, intensidade e incômodo dos sintomas angústia, ansiedade, sofrimento psicológico e função emocional deprimida apresentam pior qualidade de vida^(32)^.

Importante também salientar que alopecia apresentou prevalência de 43%, com moderada a elevada frequência, intensidade e incômodo nas pacientes, reiterando o impacto que a queda capilar pode trazer em questões psicológicas e físicas a essas mulheres^(4)^. Ademais, sintomas como náusea, diarreia, constipação, falta de apetite e perda de peso, os quais impactam diretamente no estado nutricional do paciente, também estiveram bastante presentes, com prevalência entre 30 a 40% das pacientes.

Outros sintomas que ocorreram, com moderada frequência, intensidade e incômodo, são os gastrointestinais, como náusea, falta de apetite e vômito, os quais impactam diretamente a perda de peso das pacientes. Destaca-se que a desnutrição é um dos distúrbios mais frequentes em pacientes oncológicos, especialmente quando em tratamento antineoplásico, expondo-os a piores prognósticos e complicações clínicas^(33)^. Devido a esses sintomas, muitas pacientes deixam de se alimentar, e apresentam inapetência ou saciedade precoce, ocasionando, como consequência, o emagrecimento, podendo evoluir para um quadro mais grave: a caquexia^(33)^.

Estudo observacional transversal conduzido em CACON na região Sudeste do Brasil, que objetivou avaliar o estado nutricional de mulheres em tratamento para câncer de mama em estágios I, II ou III, encontrou associações significativas entre risco nutricional e nível educacional (p=0,03) e Índice de Massa Corporal (IMC) (p=0,01). A análise da regressão logística binária revelou associação significativa entre nível educacional e risco nutricional, indicando que menor nível educacional foi associado a maiores chances de risco nutricional (OR = 4,59; IC95% = 1,01-21,04; p=0,049). Além disso, em relação ao IMC, observou-se que um IMC acima de 20,5 kg/m^2^ foi associado a maior probabilidade de risco nutricional (OR = 0,09; IC95% = 0,01-0,89; p=0,039)^(34)^. Entretanto, em outro estudo, foi evidenciado que marcadores inflamatórios, como a razão neutrófilos-linfócitos e a razão plaquetas-linfócitos de pacientes com câncer de mama não metastático hospitalizadas, não apresentaram associação significativa com fatores nutricionais^(35)^.

Recente estudo que objetivou avaliar, comparar o estado nutricional de mulheres com câncer de mama em estágios I a III no primeiro e terceiro ciclos de quimioterapia ambulatorial e identificar fatores associados evidenciou que o sobrepeso foi predominante em ambos os ciclos de quimioterapia. Aproximadamente 6,67% e 10% das pacientes apresentaram risco nutricional no 1º e 3º ciclos de quimioterapia, respectivamente. Ansiedade e depressão foram prevalentes no 1º ciclo de quimioterapia, e foram significativamente associadas ao risco nutricional (p= 0,002). As variáveis idade no 3º ciclo e dor/desconforto no 1º ciclo (p=0,049 e p=0,043, respectivamente) apresentaram associação significativa com o risco nutricional^(36)^.

### Limitações do estudo

O estudo apresenta algumas limitações. Tratam-se de dados coletados em um único centro de referência em oncologia do Brasil, com um pequeno tamanho amostral. Ademais, o desenho observacional descritivo não permite associações bivariadas e multivariadas. Outro limitador foi incluir pacientes com câncer de mama não metastático em estágios I, II e III, com diferentes protocolos quimioterápicos e em qualquer fase do tratamento antineoplásico.

### Contribuições para a área da enfermagem

É crucial uma melhor compreensão sobre como o padrão de *clusters* de sintomas oncológicos se configuram em mulheres com câncer de mama não metastático hospitalizadas, de modo a possibilitar a elaboração de intervenções personalizadas que possam contribuir para uma melhor assistência a essas pacientes, com impacto positivo na sua qualidade de vida^(37-40)^. A atuação de uma equipe multidisciplinar e interprofissional se faz necessária para o manejo dos *clusters* de sintomas oncológicos apresentados por essas pacientes, por meio de um cuidado personalizado e centrado nas preferências de cada paciente.

## CONCLUSÕES

O *cluster* de sintoma neuropsicológico 3 (dor, fadiga, preocupações e distúrbio do sono) foi o mais prevalente em mulheres hospitalizadas com neoplasia maligna da mama não metastático.
